# Biophysical and structural characterization of a multifunctional viral genome packaging motor

**DOI:** 10.1093/nar/gkad1135

**Published:** 2023-12-12

**Authors:** Nikolai S Prokhorov, Christal R Davis, Kashyap Maruthi, Qin Yang, Michael B Sherman, Michael Woodson, Mark A White, Lohra M Miller, Martin F Jarrold, Carlos E Catalano, Marc C Morais

**Affiliations:** Department of Biochemistry and Molecular Biology, The University of Texas Medical Branch at Galveston, Galveston, TX 77555, USA; Sealy Center for Structural Biology, The University of Texas Medical Branch at Galveston, Galveston, TX 77555, USA; Department of Molecular and Cellular Biochemistry, Indiana University, Bloomington, IN 47405, USA; Program in Structural Biology and Biochemistry, University of Colorado Anschutz Medical Campus, Aurora, CO 80045, USA; Department of Biochemistry and Molecular Biology, The University of Texas Medical Branch at Galveston, Galveston, TX 77555, USA; Sealy Center for Structural Biology, The University of Texas Medical Branch at Galveston, Galveston, TX 77555, USA; Department of Pharmaceutical Chemistry, Skaggs School of Pharmacy and Pharmaceutical Sciences, University of Colorado Anschutz Campus, Aurora, CO 80045, USA; Department of Biochemistry and Molecular Biology, The University of Texas Medical Branch at Galveston, Galveston, TX 77555, USA; Sealy Center for Structural Biology, The University of Texas Medical Branch at Galveston, Galveston, TX 77555, USA; Sealy Center for Structural Biology, The University of Texas Medical Branch at Galveston, Galveston, TX 77555, USA; Department of Biochemistry and Molecular Biology, The University of Texas Medical Branch at Galveston, Galveston, TX 77555, USA; Sealy Center for Structural Biology, The University of Texas Medical Branch at Galveston, Galveston, TX 77555, USA; Department of Chemistry, Indiana University, Bloomington, IN 47405, USA; Department of Chemistry, Indiana University, Bloomington, IN 47405, USA; Program in Structural Biology and Biochemistry, University of Colorado Anschutz Medical Campus, Aurora, CO 80045, USA; Department of Pharmaceutical Chemistry, Skaggs School of Pharmacy and Pharmaceutical Sciences, University of Colorado Anschutz Campus, Aurora, CO 80045, USA; Department of Biochemistry and Molecular Biology, The University of Texas Medical Branch at Galveston, Galveston, TX 77555, USA; Sealy Center for Structural Biology, The University of Texas Medical Branch at Galveston, Galveston, TX 77555, USA; Department of Molecular and Cellular Biochemistry, Indiana University, Bloomington, IN 47405, USA

## Abstract

The large dsDNA viruses replicate their DNA as concatemers consisting of multiple covalently linked genomes. Genome packaging is catalyzed by a **terminase enzyme** that excises individual genomes from concatemers and packages them into preassembled procapsids. These disparate tasks are catalyzed by terminase alternating between two distinct states—a stable nuclease that excises individual genomes and a dynamic motor that translocates DNA into the procapsid. It was proposed that bacteriophage λ terminase assembles as an anti-parallel dimer-of-dimers nuclease complex at the packaging initiation site. In contrast, all characterized packaging motors are composed of five terminase subunits bound to the procapsid in a parallel orientation. Here, we describe biophysical and structural characterization of the λ holoenzyme complex assembled in solution. Analytical ultracentrifugation, small angle X-ray scattering, and native mass spectrometry indicate that 5 subunits assemble a cone-shaped terminase complex. Classification of cryoEM images reveals starfish-like rings with skewed pentameric symmetry and one special subunit. We propose a model wherein nuclease domains of two subunits alternate between a dimeric head-to-head arrangement for genome maturation and a fully parallel arrangement during genome packaging. Given that genome packaging is strongly conserved in both prokaryotic and eukaryotic viruses, the results have broad biological implications.

## Graphical Abstract

**Graphical Abstract:**
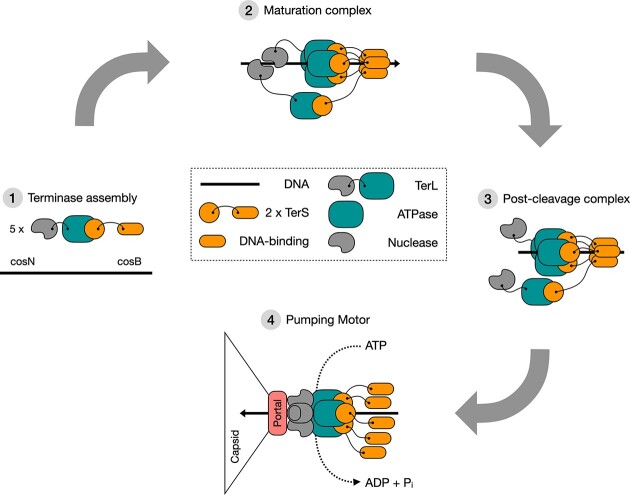

## Introduction

**Figure 1.**
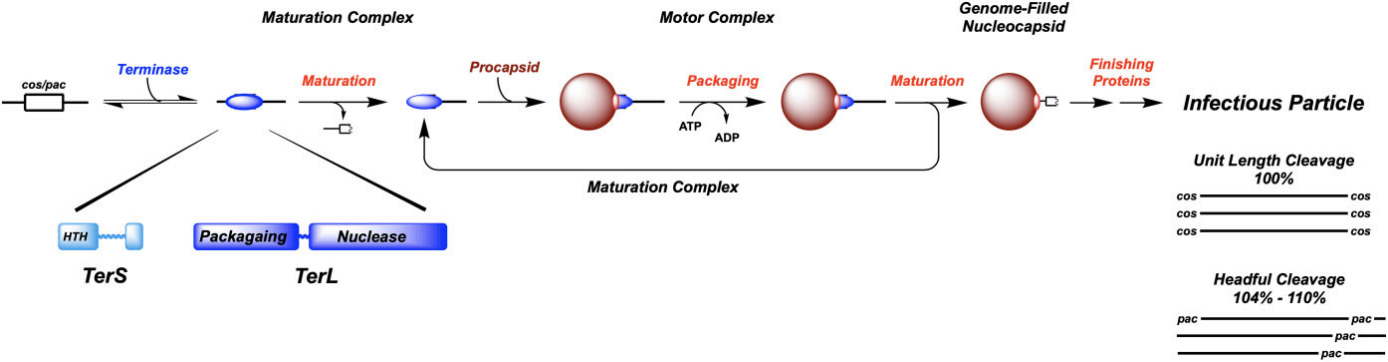
Genome Packaging in the Complex Double-Stranded DNA Viruses. Terminase enzymes are responsible for processive excision of an individual genome from a concatemeric packaging substrate (genome maturation) and for translocation of the duplex into a pre-formed procapsid shell (genome packaging). The functional enzymes are composed of large catalytic (TerL) and small DNA recognition (TerS) subunits, both of which possess conserved functional domains, as depicted at bottom, *left*. Two basic strategies for genome packaging, unit-length and headful, are summarized at bottom, *right*. Processive packaging requires that terminase cycles between a maturation complex, tightly bound at a *pac* or *cos* site in the viral genome, and a dynamic motor complex that processively packages DNA. Details are provided in the text.

Viruses are obligate intracellular parasites whose developmental pathways are initiated upon insertion of their genetic material into a host cell (1). The pathways are generally conserved within broad virus classes such as the large dsDNA viruses, including, the Caudoviruses (tailed bacteriophages) and the Herpesviruses groups (2,3). These viruses typically replicate their genomes as covalently linked head-to-tail concatemers (immature DNA). Expression of late viral genes produces structural proteins that self-assemble into procapsid shells into which the newly replicated DNA is actively packaged. Genome-packaging, which is catalyzed by terminase enzymes, represents the intersection between the DNA replication and capsid assembly pathways (4–7); this involves processive excision of individual genomes from the concatemer and simultaneous packaging of the ‘mature’ genome into a preassembled procapsid shell (Figure 1). Both reactions are catalyzed by virus-encoded terminase enzymes, which thus perform two essential functions: (i) nucleolytic excision of individual genomes from a concatemeric precursor (maturation reaction) and (ii) concomitant ATP hydrolysis-coupled translocation of viral DNA (packaging reaction). To accomplish these distinct tasks, terminase complexes alternate between stable maturation and dynamic motor configurations.

There are two basic strategies for processive genome packaging from a concatemeric DNA precursor. The first is ‘headful’ packaging (phages T4, P22, SPP1, P74-26, etc.) wherein terminase site-specifically assembles at a packaging initiation site (*pac*) and cuts the duplex to form the first genome end to be packaged. This maturation complex transitions to a dynamic motor complex that translocates DNA into the procapsid shell (a.k.a., head). Packaging continues *past* the next downstream *pac* site until the shell is filled to capacity. At this point, the motor stops and transitions back to a nuclease complex that again cuts the duplex to release the nucleocapsid. In this case, the capsid is filled with 104–110% genome length of DNA as depicted in Figure 1. The second strategy is exemplified by phages λ, HK97 and the herpesviruses that package ‘unit-length’ genomes. In this case, the maturation complexes assemble at a *cos* sequence in the concatemer, cut the duplex, bind to a procapsid and transition to a motor complex that packages the DNA. Unlike the headful phages, however, the motor is captured by the next downstream *cos* sequence and duplex nicking releases the nucleocapsid filled with a unit length (100%) mature genome (Figure 1). Notable exceptions to this general paradigm are represented by the ϕ29-like bacteriophages and the adenovirus groups, which replicate monomeric genomes in a protein-primed manner and thus have no maturation requirement. Notwithstanding, these viruses utilize an analogous ‘packaging ATPase’ enzyme to package viral DNA into a pre-assembled procapsid shell.

Terminase enzymes function as heterooligomeric complexes (holoenzymes) consisting of a large catalytic subunit (TerL) that contains both maturation (nuclease) and packaging (ATPase) activities and a small DNA recognition subunit (TerS) that is required for site-specific assembly at the packaging initiation site (Figure 1) (4–7); both subunits are essential for virus development *in vivo* (6,8–10). With a few exceptions (11,12), characterized isolated TerL subunits are monomers in solution, but assemble as a functional pentameric motor at the portal vertex of a procapsid (5,7,13). In contrast, isolated TerS subunits assemble into ring-like complexes of varying stoichiometries and central channel dimensions, and it is presumed that these rings reflect the assembly state of TerS in holoenzyme during maturation at *pac*/*cos* (5,6,8). Genetic and biochemical data indicate that both subunits (the holoenzyme) are required to assemble the maturation complex to initiate genome packaging (5–7). The variability of TerS assemblies in different systems has led to a controversy as to whether DNA passes through the central channels of the assembled complexes or whether the complexes wrap the duplex around the TerS ring exterior (14–21). However, these interpretations are complicated by the fact that the TerS structures were obtained in the absence of the cognate TerL subunit which is required for the assembly of a functional complex. There is little information on the holoenzyme complexes assembled from both subunits, largely due to the challenge of recombinantly expressing and/or assembling a functional TerL•TerS holoenzyme. Exceptions are bacteriophages lambda and P22 (22–24). In lambda, terminase has been shown to assemble as a stable holoenzyme protomer consisting of one TerL and two TerS subunits (24,25).

The λ terminase protomer is devoid of catalytic activity but undergoes a salt-linked self-association reaction *in vitro* (*K_D,app_*∼ 3–4 μM) to provide a multimeric ring-like holoenzyme complex that possesses native nuclease, ATPase and DNA packaging activities (24–27). At physiological concentrations (∼100 nM), the protomer and the cellular integration host factor (IHF) cooperatively assemble at the *cos*-sequence of λ DNA (*K_D,app_*∼ 20 nM), which activates *cos*-specific nuclease activity but down-regulates the ATPase activity (26,27). Consistent with *in vivo* data, *cos*-cleavage is stimulated by ATP/ADP (5,28–31). Based on extensive biophysical data and biological precedence, we proposed that the protomers assemble as a head-to-head, anti-parallel dimer of dimers, thus orienting the maturation complex to introduce symmetric nicks into the duplex (4,5). Such an arrangement would be analogous to that seen in the type IIE and IIF restriction endonucleases (32–34).

The post-cleavage complex binds to the portal vertex of an empty procapsid, which triggers the transition to a dynamic motor complex in which nuclease activity is downregulated and ATPase activity is upregulated to power DNA packaging (Figure 1). In contrast to the proposed tetrameric λ maturation complex, biochemical, biophysical, and structural characterization of motor complexes in several phage systems indicate that they operate as *pentamers* during translocation (5), and functional pentameric packaging motors have been imaged to near-atomic resolution in ϕ29-like phages. Genetic and biochemical studies in λ, P22, T3 and T4 (5,35,36) and structural studies in ϕ29 (37) indicate that C-terminal residues of TerL interact with the portal during packaging. Thus, it has been presumed that viral packaging motors are composed of TerL subunits assembled in a pentameric ring-like complex, oriented in a *parallel* manner. This contrasts with the anti-parallel, head-to-head dimer-of-dimers λ maturation complex described above.

To reconcile these observations, we proposed that the stoichiometry and orientation of λ TerL subunits in the maturation complex (anti-parallel complex) may be distinct from that assembled in the dynamic motor complex bound to the capsid (parallel pentamer), ostensibly due to their vastly different biochemical roles in genome packaging. However, there is no structural data for the λ motor complex to verify the hypothesis. Moreover, there is no structural data for a motor or maturation complex composed of both requisite TerL and TerS subunits in any system. To fill this gap, a combination of analytical ultra-centrifugation (AUC), charge detection mass spectrometry (CDMS), small angle X-ray scattering (SAXS), enzyme kinetics, and class averaging of cryo-EM images is employed to investigate the structure and function of the λ terminase holoenzyme. The ensemble of data is consistent with a stable but highly flexible pentameric complex deviating from a strict 5-fold symmetry in a 4 + 1 fashion. The complex undergoes substantial rearrangement upon ATP binding towards a more compact conformation presumably more suitable for DNA gripping with four large terminase subunits forming an open ring and the fifth subunit exhibiting a high degree of conformational freedom. We propose a novel model wherein the dynamic nature of this ‘special subunit’ allows a single holoenzyme complex to perform both essential packaging reactions - antiparallel cleavage of a DNA target and DNA-packaging by a parallel arrangement of components. Finally, we propose a ‘symmetry resolution’ model to explain how λ terminase transitions between the distinct structural and functional states to processively excise and package multiple genomes from concatemeric DNA.

## Materials and methods

### General

The terminase protomer and ring species were purified by published procedures using a combination of immobilized metal affinity chromatography, anion-exchange chromatography, and gel filtration (38,39). The purified enzyme is >98% homogenous as determined by SDS-PAGE and the protomeric species is >98% structurally homogenous as determined by sedimentation velocity analytical ultracentrifugation analysis. Assembly of the catalytically competent terminase ring was performed as previously described (26,27,40).

### Saxs

SAXS experiments were performed at the Sealy Center for Structural and Computational Biology (SCSB) at the University of Texas Medical Branch (UTMB). The X-ray/SAXS station at the SCSB consists of a high-brilliance FR-E++DW Superbright X-ray generator, with the industry standard RAXIS-IV++ crystallography system with both Cu and Cr optics. In addition, the source is connected to a Rigaku BioSAXS-1000 with a Kratky camera and a 96-well automatic sample changer. Samples were illuminated with X-rays generated by the Cu anode, corresponding to the wavelength of 1.5418 Å. Scattering intensities I(q) for the protein and buffer samples were recorded as a function of scattering vector *q* (*q* = 4πsinθ/λ, where 2θ is the scattering angle and λ is the X-ray wavelength). The sample-to-detector distance was 0.476 m, which resulted in a q range of 0.009–0.68 Å^−1^, and all experiments were performed at 5°C. The data collection strategy described by Hura was used in this study (41). Briefly, SAXS data were collected for three protein concentrations (0.80, 0.50 and 0.25 mg/ml) and for three matching buffer samples. For each sample measurement, SAXS data were collected with 1-hour sub-frames to assess radiation damage. The buffer scattering contributions were subtracted from the sample scattering data using the SAXNS_ES web server (https://xray.utmb.edu/SAXNS/). Data analysis was performed using the program package PRIMUS from the ATSAS suite (42,43). Experimental SAXS data obtained for different protein concentrations were analyzed for aggregation and folding state using Guinier and Kratky plots, respectively. The forward scattering intensity *I*(0) and the radius of gyration *R*_g_ were evaluated using the Guinier approximation: *I*(*q*) ≈ *I*(0) exp(−*q*2*R*_g_)2/3, with the limits *qR*_g_ <1.5. These parameters were also determined from the pair-distance distribution function *P*(*r*), which was calculated from the entire scattering patterns via indirect Fourier inversion of the scattering intensity *I*(*q*) using the program GNOM (44). The maximum particle diameter Dmax was also estimated from the *P*(*r*). The hydrated volume VP of the particle was computed using the Porod equation: VP = 2π2*I*(0)/*Q*, where *I*(0) is the extrapolated scattering intensity at zero angle and *Q* is the Porod invariant. (45,46). The molecular mass of a globular protein can then be estimated from the value of its hydrated volume following the method of Rambo and Tainer as implemented in the SAXNS_ES server (45). The overall shape of the protein was modeled *ab initio* by fitting the SAXS data to the calculated SAXS profile of a chain-like ensemble of dummy residues in reciprocal space using the program GASBOR, version 2.3i (47). Twenty-five independent calculations were performed with a D5 symmetry restriction (see below for choice of symmetry).

### Analytical ultracentrifugation (AUC)

Analytical ultracentrifugation experiments were performed using an Optima XL-A analytical ultracentrifuge equipped with absorbance optics (Beckman Coulter, Brea, CA). The purified terminase ring was dialyzed into 20 mM Tris–HCl buffer, pH 8 at 4°C containing 500 mM NaCl, 5% v/v glycerol and 1 mM TCEP and then concentrated using 10 kDa MWCO Amicon Centrifugal Filters (Millipore Sigma) according to manufacturer's directions. The enzyme concentration was determined spectrophotometrically (ϵ280 = 179 680 cm^−1^ M^−1^ for the protomer) and then diluted using the dialysis buffer to the concentration indicated in each individual experiment. Buffer viscosity (1.893 mPa*s) and buffer density (1.03812 g/cm^3^) were measured at 4°C using a Lovis 2000 M rolling ball viscometer/densitometer (Anton Paar). A $\bar{\nu }$ of 0.72364 ml/g at 4 °C and 0.73045 ml/g at 20°C was determined using the Sedntrp program (Biomolecular Interactions Technology Center, University of New Hampshire, Durham, NH) and corrected for the change in hydration shell due to the presence of glycerol to yield 0.72397 ml/g at 4 °C and 0.73078 ml/g at 20°C as described elsewhere.

For the *sedimentation velocity e*xperiments (SV-AUC), the samples (410 ml) were loaded into the sample cell of two sector Epon charcoal-filled centerpieces and dialysis buffer was loaded into the reference cell. The samples were allowed to equilibrate to 4°C and then spun at 32 000 rpm; sedimentation was monitored by absorbance at 250 nm. The data were analyzed using the SEDFIT program to afford c(s) distributions (48). The bottom fitting limit was moved to 6.7 cm to improve the fit accuracy by avoiding the glycerol gradient created at the cell bottom as reported elsewhere.

For the *sedimentation equilibrium* experiments (SE-AUC), 80 μl of each sample at the indicated concentration was loaded into the reference cell of six sector Epon-filled charcoal centerpieces and dialysis buffer was loaded into the reference cell. The samples were allowed to equilibrate to 4°C and then successively spun at 7500, 9000 and then 11 000 rpm. Sedimentation at each speed was assumed to be at equilibrium when consecutive scans, separated by intervals of 2 h, did not change as determined using the Heteroanalysis program (49). Absorbance data were collected at 250 nm every 0.003 cm in the step mode, with 5 averages per step. The protomer e250 (69 373 M^−1^cm^−1^) was determined using a concentration curve and fit using a linear least squares approach (see Figure SX). A global non-linear, least-squares (NLLS) analysis of the combined SE data was performed using the SedAnal program (50) assuming a single-species model;


\begin{equation*} {\mathrm{c}}_{\mathrm{r}} = \mathop \sum \limits_{{\mathrm{i}} = 1}^{\mathrm{n}} {{\mathrm{c}}}_{{\mathrm{bi}}}{\mathrm{exp}}\left[ {\frac{{\left( {1- {{\rm\nu{}}}_{\mathrm{i}}{\rm\rho{}}} \right){{\rm\omega{}}}^2{{\mathrm{M}}}_{\mathrm{i}}\left( {{{\mathrm{r}}}^2 - {\mathrm{r}}_{\mathrm{b}}^2} \right)}}{{2{\mathrm{RT}}}}} \right] + {\mathrm{b}}\end{equation*}

where *c*_bi_, *v*_i_, and *M*_i_ are the concentration at the bottom of the cell, partial specific volume and molecular mass of the ‘I’ component, respectively; ρ is the density of the solution; ω is the angular velocity; and *b* is the base-line error term.

### Mass spectrometry

The terminase samples were exchanged into ammonium acetate solution, a volatile salt used in native electrospray, by Zeba microbiospin columns, 7 kDa MWCO (Thermo Fisher Scientific). Terminase protomer was exchanged into a 200 mM ammonium acetate salt, and assembled ring was exchanged into a 300 mM ammonium acetate salt pH 8.34. After buffer exchange samples were immediately electrosprayed for CDMS analysis.

Charge detection mass spectrometry (CDMS) measurements were performed on a home-built instrument described in detail elsewhere (51–58). Briefly, the mass to charge ratio (*m/z)* and charge are simultaneously measured on thousands of individual particles and binned to give a mass distribution of the sample. Ions enter the instrument through a metal capillary after formation from a nanoelectrospray (nESI) source. The positively charged ions are guided through a series of ion optics and differential pumping to an electrostatic linear ion trap (ELIT) where they oscillate back and forth through a detection cylinder. Signal from the oscillating ion is picked up on a charge sensitive amplifier where it is digitized and processed using a fast Fourier transform. The fundamental frequency measured corresponds to the *m/z* of the ion and the magnitude corresponds to the charge.

### cryoEM methods

Quantifoil R2/1 holey carbon copper grids (Electron Microscopy Sciences, Hatfield, PA, USA) were plasma-cleaned for 40 s in Solarus plasma cleaner (Gatan, Inc.) using H_2_O_2_ plasma. A total of 2.5 μl of purified terminase at 0.5 mg/ml was applied to the grid prior to freezing using a Vitrobot IV (Thermo Fisher Scientific) automated plunge-freezing robot. For the ATPγS stalled sample, ATPγS was added to get a 1 mM final concentration prior to freezing. Grids were loaded into a Titan Krios G3i microscope (Thermo Fisher Scientific) housed at SCSB center at UTMB, and 500 micrographs for each sample were collected at 300 keV on a Falcon III direct electron detector operating in linear mode at 75K nominal magnification at the detector, thus corresponding to a pixel size of 1.1 Å/pixel. The defocus range was set from −1.4 to −3.2 μm. Each area was exposed to a total dose of 50 electrons/Å^2^ in 0.97 s. 38 fractions were recorded per exposure. Collected movie fractions were processed in CryoSPARC v4.2 (59). Both datasets contained ∼244 particles per micrograph on average. 18 925 particles of apo terminase and 24 408 particles of ATPγS stalled terminase went into well-aligned 2D classes after several rounds of classification.

### Structure prediction

A full implementation of Alphafold2 installed at the Texas Advanced Computing Center was used with default settings to generate computed structure models (CSMs) of the terminase protomer (60).

### ATPase kinetic analysis

ATPase assays were performed by published procedure (61,62), with modification. Briefly, reaction mixtures (10 μl) contained 50 mM Tris–HCl, pH 9, 10 mM MgCl_2_, 60 mM NaCl, 2 mM spermidine, 7 mM β-ME, 5 μM [α^32^P] ATP, and 10 nM lambda terminase. The reaction mixtures were incubated at 37°C for 5 min and were initiated with the addition of terminase and allowed to proceed for 20 min at 37°C. Aliquots (2 μl) were removed from the reaction mixture and quenched with the addition of equal volume stop solution (100 mM EDTA, 10 mM each cold ATP and ADP). Aliquots (2 μl) of the quenched reaction mixtures were spotted onto a silica gel TLC plate and the plate was developed with 60% of 2-propanol and 30% ammonium hydroxide. The images of ^32^P labeled ATP and ADP spots were captured by GE-Typhoon Phosphorimager and quantitated using the ImageQuant software. Control experiments (ATP hydrolysis time course) using these conditions were performed to ensure that the aliquots were taken within the linear portion of the reaction curve. The kinetic constants for ATP hydrolysis were determined by non-linear regression analysis of the experimental data using the Igor data analysis program (Wave Metrics, Lake Oswego, OR).

## Results

### Small-angle X-ray scattering

**Figure 2.**
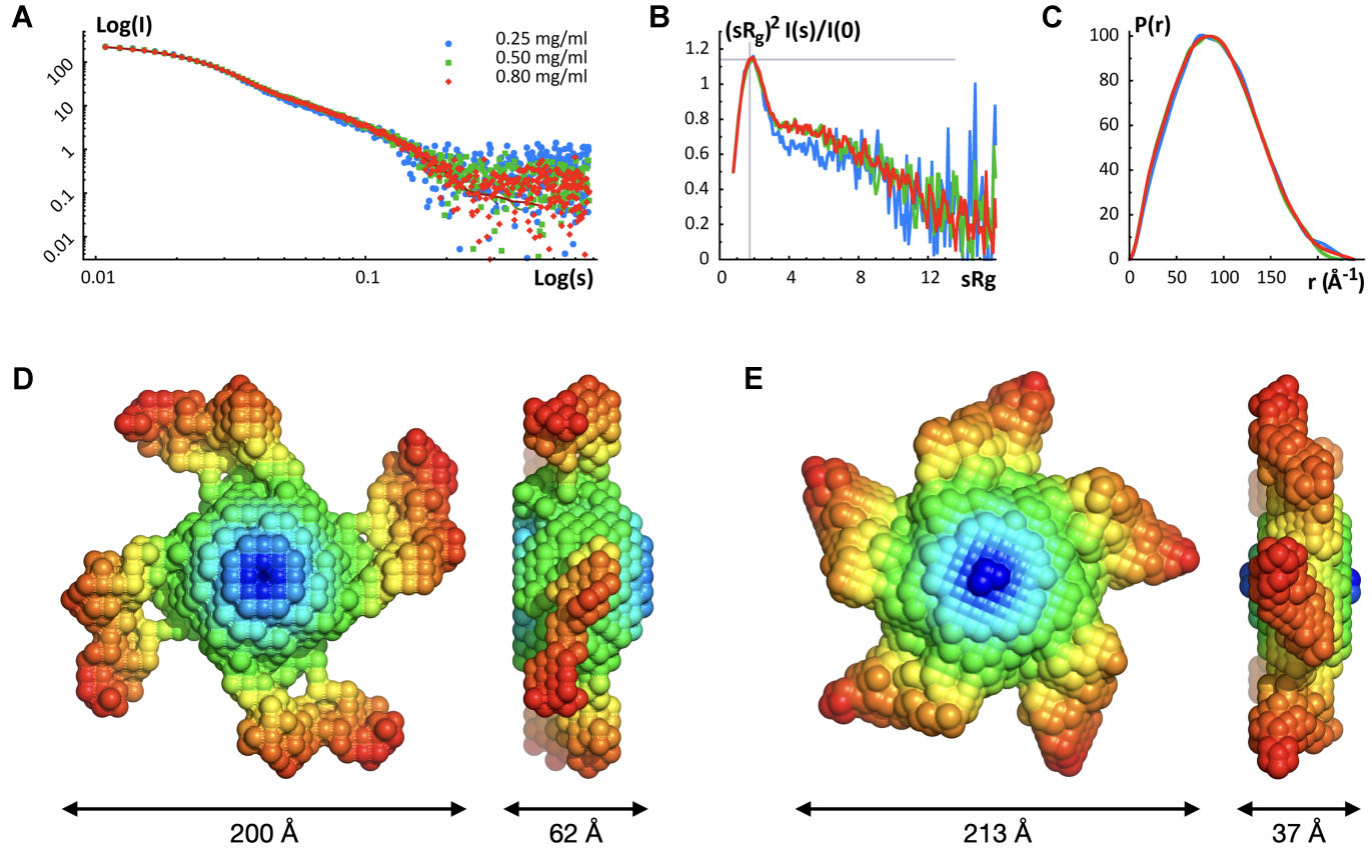
SAXS analysis of the assembled terminase holoenzyme. Panel **A**. Experimental scattering patterns for three concentrations of λ terminase scaled to concentration. Kratky plots (Panel **B**) and pair-distance distance distribution functions *P*(*r*) (Panel **C**) of the assembled λ terminase ring for the three concentrations. Panels D and E. Molecular envelopes generated using GASBOR. Models were obtained by imposing either P4 (Panel **D**) or P5 (Panel **E**) symmetry. Both models are equally consistent with the X-ray scattering curve of terminase holoenzyme.

As a first step towards deciphering size and shape information for λ holoenzyme, we conducted solution X-ray scattering experiments on λ protomers assembled into the catalytically competent ring complex. No radiation damage in the assembled complexes was detected over the 10–12-h exposure time, so the data obtained after the long exposure was used for analysis. The scattering profiles are independent of the protein concentration, as the scattering curves superimposed well for protein samples at concentrations ranging from 0.25 to 0.80 mg/ml (Figure 2A). This is consistent with our previous AUC data demonstrating that a single homogeneous species, the assembled ring, predominates in this concentration range (vide infra) (26,27).

Kratky plots show that the complex is well-folded overall but indicate that there are likely small regions of polypeptide that are not well ordered (Figure 2B). Two independent methods, the Guinier approximation and the pair-distribution function P(r), were used to calculate the radius of gyration (*R*_g_) value for each protein concentration. Both methods provide similar *R*_g_ values, with ≈70 ± 1 Å calculated from the Guinier approximation and ≈71.9 ± 0.6 Å estimated from the pair-distance distribution function *P*(*r*) (Table 1). The maximum dimension of the particle, *D*_max_, was found to be ≈230 Å by examining where the pair-distance distribution function went to zero (Figure 2C). The molecular weight determined using the Porod-invariant volume method corresponded to average molecular masses between 479.7 and 586.7 kDa indicating that terminase forms a higher order assembly in solution consisting of ∼4.6 protomers, on average (Table 1).

**Table 1.** SAXS parameters for the assembled terminase holoenzyme

Concentration (mg/ml)	Guinier *R*_g_ (Å)	*D*_max_ (Å)	*P*(*r*) *R*_g_ (Å)	Mass (kDa)	Stoichiometry
0.25	71.2 (23)	241	73.0 (1)	586.7	5.1
0.50	74.1(24)	228	71.1 (8)	479.7	4.2
0.80	70.5 (12)	233	71.9 (6)	503.8	4.4

### SAXS *ab initio* shape calculations

The shape of the assembled terminase holoenzyme in solution can be approximated from the shape of the Kratky plot (Figure 2B) and the pair-distance distribution function (*P*(*r*), Figure 2C). The Kratky plot shows a bell-shaped peak at low angles, which indicates a well-folded protein, and *P*(*r*) shows a characteristic shape of a conical particle (45,63,64).

Bead models of the assembly, which approximate the molecular envelope, were then obtained using the program GASBOR. Due to the non-integer number of subunits indicated by the Porod volumes calculated at different protein concentrations (average of 4.6 protomers), GASBOR was run imposing either P4 or P5 symmetry on the resulting bead model. Models with P4 and P5 symmetry had similar dimensions, and both were conical ring-like shapes with similar dimensions. The ‘base’ of the cone had a diameter of ≈200–213 Å and the height of the cones was ∼37–62 Å (Figure 2D and E). Calculated scattering curves from each of the 25 envelopes with either P4 or P5 symmetry imposed fit the experimental scattering data equally well, with ∼χ^2^ values ranging from 0.82 to 0.89; models for either symmetry did not cluster within this range, and hence the ∼χ^2^ values could not be used to distinguish between potential stoichiometries.

### Hydrodynamic modeling of the terminase ring

We previously employed analytical ultracentrifugation (**AUC**) to evaluate the protomer stoichiometry in the assembled lambda maturation complex and we interpreted these data to indicate that the holoenzyme is composed of four protomers assembled into a ring-like structure (25,26). However, the SAXS results above are consistent with either a tetrameric or pentameric ring complex. To correlate the two studies, we modeled the hydrodynamic behavior of the two SAXS models using the HYDROPRO program (65). The data presented in Table 2 show that the predicted sedimentation coefficient of the pentameric ring (13.6 S) most closely matches the previously determined sedimentation coefficient of the terminase ring (26).

**Table 2.** Hydrodynamic modeling of the terminase holoenzyme SAXS data

	Tetramer ring	Pentamer ring
Molecular weight (calculated)	460 kDa	575 kDa
Radius of gyration	7.4 × 10^−7^ cm	7.3 × 10^−7^ cm
Volume	6.5 × 10^−19^ cm^3^	7.4 × 10^−19^ cm^3^
*s_(20,w)_*	11.8 S	13.6 S

### Biophysical characterization of terminase holoenzyme

**Figure 3.**
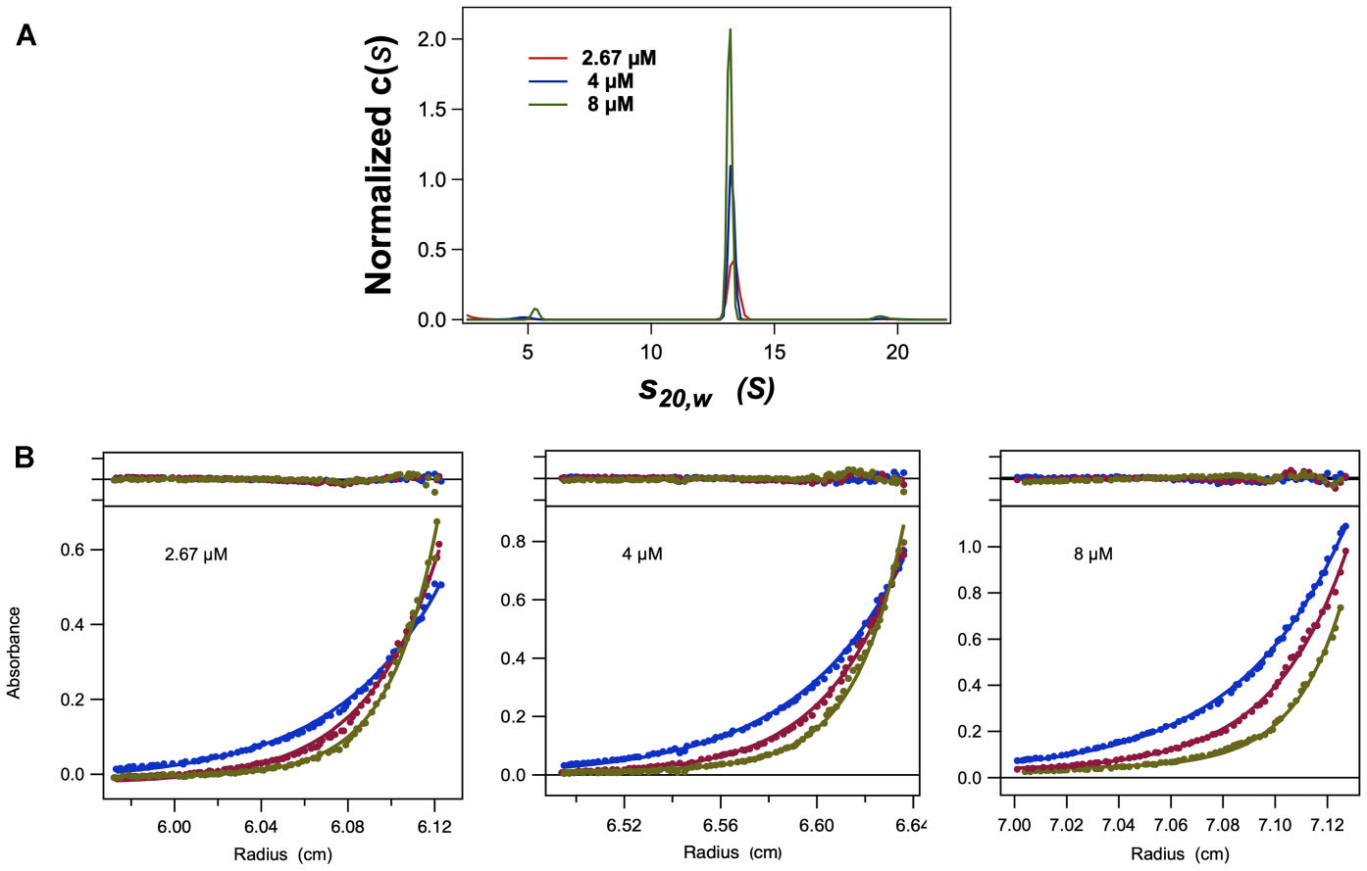
Analytical ultracentrifugation of the assembled terminase holoenzyme. Panel **A**. Normalized *c*(*s*) distribution of the SV-AUC data analyzed by Sedfit as described in Materials and Methods. Note the appearance of minor 5.1 *s* and 19.3 *s* species, which represent a small fraction (<5%) of the ring that has dissociated to the protomer and presumed ring dimers, respectively. Panel **B**. Sedimentation equilibrium analysis of the assembled terminase holoenzyme. Sample concentrations were 2.67, 4 and 8 μM as indicated and were spun at 7500 rpm, 9000 rpm, and 11 000 rpm as described in Materials and Methods.

Given that the protomer stoichiometry has important mechanistic implications in both the maturation and motor complexes, we re-examined the protomer stoichiometry in the assembled holoenzyme using an expanded AUC approach. We first employed sedimentation velocity analytical ultracentrifugation (SV-AUC) as described in Materials and Methods. Three different concentrations of protomer (2.67 μM–0.31 mg/ml, 4 μM–0.46 mg/ml and 8 μM–0.92 mg/ml, similar to those used in the SAXS studies) were examined and the data was analyzed using three independent approaches. First, the model-independent time derivative approach (DCDT^+^) was employed, and the data are consistent with a single apparent species. Analysis of the g*(s) distributions affords an *s*(_20,*w*_) of 13.1 to 13.5 S for the three protein concentrations (Table 3). We next used Sedfit, a direct boundary fitting approach, and the *c*(*s*) distributions are shown in Figure 3A. Similar to the DCDT^+^ results, a single predominant species is observed, and the analysis yields *s*(_20,*w*_) of 13.2 to 13.3 S for the three protein concentrations (Table 3). Finally, we employed SEDANAL to simultaneously fit all three data sets according to a single species model. The results of the global fit yield *s*(_20,*w*_) = 13.6 S (Table 3). In sum, the three analytical approaches afford similar values for *s*(_20,*w*_) that are commensurate with the hydrodynamic modeling of the SAXS data (Table 2) and our published data (25–27). However, the calculated protomer stoichiometry of the holoenzyme differs depending on which analytical approach was employed, ranging from 3.3 to 4.4 protomers per assembly (Table 3).

**Table 3.** AUC analysis of the assembled terminase holoenzyme ring

	Speed (rpm)	Method	*s* *_(20,w)_*	Derived MW (kDa)	Derived stoichiometry*
*SV-AUC*					
2.67 mM	32 k	DCDT+	13.5	503	4.4
4 mM	32 k	DCDT+	13.4	477	3.9
8 mM	32 k	DCDT+	13.1	481	3.6
2.67 mM	32 k	SEDFIT	13.3	446	4.1
4 mM	32 k	SEDFIT	13.3	437	3.8
8 mM	32 k	SEDFIT	13.2	471	3.6
2.67 mM	32 k	SEDANAL	13.6	416	4.2
4 mM	32 k	SEDANAL	13.6	411	4.1
8 mM	32 k	SEDANAL	13.6	375	3.3
Global	32 k	SEDANAL	13.6	384	3.3
*SE-AUC*					
2.67 mM	7.5, 9, 11k		N/A	538.3	4.7
4 mM	7.5, 9, 11k		N/A	503.2	4.4
8 mM	7.5, 9, 11k		N/A	509.8	4.4
Global	7.5, 9, 11k		N/A	513.5	4.5

Thus, we next employed sedimentation equilibrium analytical ultracentrifugation (SE-AUC) to obtain the molecular weight of the assembled terminase holoenzyme that is uncluttered by shape information inherent in an SV-AUC experiment. The SE-AUC experiment used the same three protein concentrations employed in the SV-AUC studies (2.76, 4 and 8 μM) and the samples were spun at 7500, 9000 and 11 000 RPMs as described in Materials and Methods. Based on the SV-AUC results, the data were fit according to a single species model, and a global fit of all 9 data sets (three terminase concentrations and three rotor speeds) was performed; the results are presented as solid lines superimposed on the data (Figure 3B). This analysis yields a molecular weight of 513.5 kDa, which corresponds to a protomer stoichiometry of 4.5 subunits (Table 3) in agreement with the SAXS analysis.

### Characterization of terminase holoenzyme by mass spectrometry

**Figure 4.**
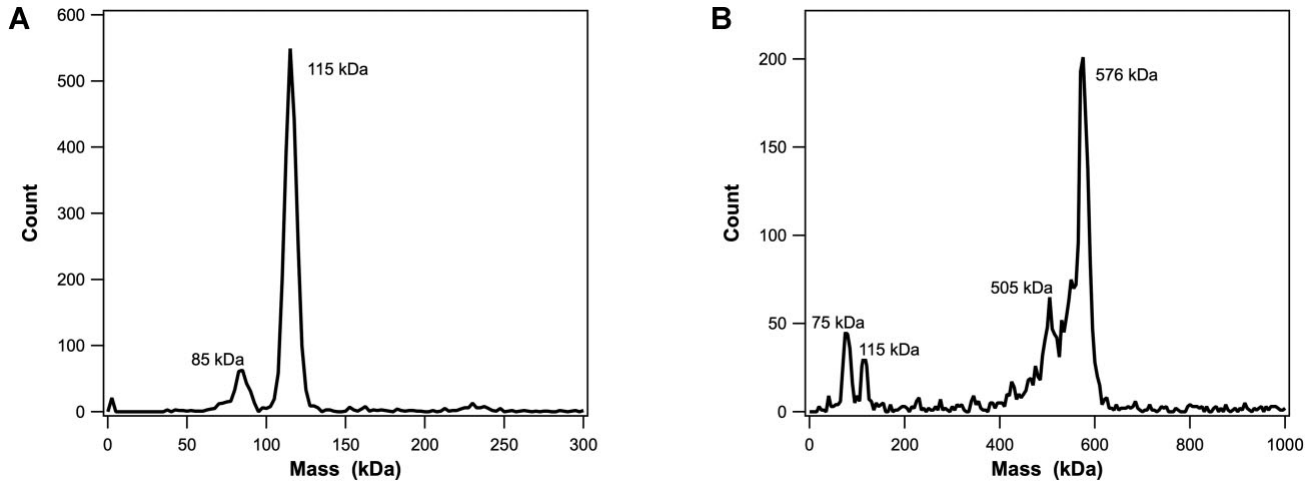
Charge detection mass spectrometry (CDMS). Panel **A**. CDMS spectra of the terminase protomer. A bin size of 5 kDa was used for the mass distribution. Gaussian fitting of the major peak gives a molecular weight of 115.434 kDa. The peaks at 85 and 230 kDa likely represent a small amount of dissociated TerL subunit and a dimer of protomers, respectively. Panel **B**. CDMS spectra of the terminase ring species. A bin size of 5 kDa was used for the mass distribution. Gaussian fitting of the major peak gives a molecular weight of 576.332 kDa. The peaks at 75 and 505 kDa likely represent an isolated TerL subunit that has dissociated from the pentamer ring and the rest of the ring, respectively. The peaks at 115 and 1130 kDa likely represent free protomer and a dimer of pentamer rings (see Figure 3A).

Charge detection mass spectrometry (CDMS) was employed to characterize the terminase protomer and assembled ring species, as described in Materials and Methods. The data for the protomer demonstrates the presence of a prominent species with a molecular weight of 115.3 kDa (Figure 4A), consistent with the molecular weight calculated from the gene sequence (115.0 kDa) and that obtained experimentally from AUC (115 ± 3 kDa) (25). CDMS analysis of the assembled ring similarly shows a predominant species with an average molecular weight of 576.3 kDa, consistent with a ring composed of 5.0 protomers (Figure 4B). A minor species with a molecular weight of 115 kDa likely represents a small amount of protomer that is in slow equilibrium with the assembled ring (25–27), similarly observed in the AUC data (see Figure 3A). Additional minor species of 505.0 and 75.0 kDa are consistent with the loss of one TerL subunit (74.1 kDa by gene sequence) from the complex. This suggests that one large subunit is less tightly bound in the assembled ring.

### CryoEM and image processing

**Figure 5.**
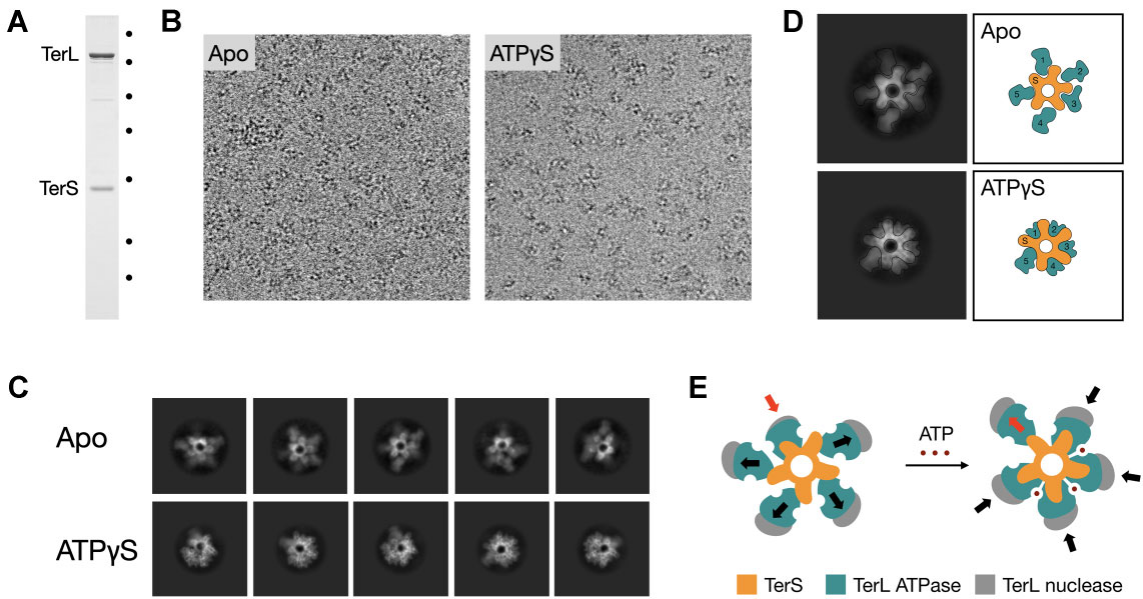
CryoEM of terminase complex. Panel **A**. SDS-PAGE of the terminase holoenzyme sample prepared for cryoEM. Marker positions are indicated on the right (14.4, 18.4, 25, 35, 45, 66.2, 116 kDa). Panel **B**. Typical cryoEM micrographs of the terminase translocation complex showing mostly end-on views of particles. The nominal magnification is 75 000×. Panel **C**. Class averages of imaged terminase particles with no ligand (apo) and ATPγS, respectively. The classes contain from a thousand to several thousand particles per class. The relative contrast is reversed in panels B and C. Note that the density corresponding to one of the five arms is weaker in most of the classes. Panel **D**. Interpretation of the 2D density in terms of the subunit organization. Panel **E**. Schematic of the ATP-driven conformational change in the terminase holoenzyme. Arrows show extended and retracted positions of TerL subunits.

To obtain further insights regarding the stoichiometry and structure of the assembled ring, we employed cryoEM. Images of terminase holoenzyme show a collection of isometric particles with a radius of ∼185 Å (Figure 5). Most particles appear to be end-on views of ring-like structures, with occasional longer conical, presumably side views with approximate dimensions of 130 Å long and ∼190 Å at the widest point. The observed dimensions of side and top views are consistent with the SAXS data and generated envelopes. Two-dimensional class averages of terminase holoenzyme confirmed preferred orientations, corresponding to end-on views of the ring. Due to the preponderance of end-on views, the paucity of particles in other orientations, and the reduced dimensionality of particle projections observed in cryoEM, it was not possible to reconstruct a 3D volume because reliable information regarding the dimension corresponding to the cone height was missing. Nonetheless, the class averages of the end-on views were of high quality and provided meaningful information. Rather than forming a continuous cone, the terminase ring more closely resembles a conical starfish-like structure with extended flexible arms, wherein the apex of the cone, the central disk of a starfish, likely corresponds to the small terminase ring (Figure 5). Consistent with a pentameric stoichiometry, the class averages show five radial extensions corresponding to starfish-like arms, which we interpret as dynamic TerL subunits. It is worth noting that while the particles are clearly pentameric, most classes deviate from 5-fold symmetry. In these classes, one or two arms are not related to their neighbors by a strict 72-degree rotation, and sometimes appear shortened/differently oriented, with weaker more diffuse electron density. The class averages show a central pore of ∼29 Å, confirming that these higher order terminase assemblies are ring-like structures, consistent with other phage terminases and ASCE ring ATPase motors (66).

Biochemical studies have demonstrated that adenosine nucleotides modulate terminase DNA-binding interactions and stimulate the nuclease activity of the protomer (5,28–31). Therefore, we imaged the λ terminase ring bound to ATP-γS, a poorly hydrolyzed ATP analog. These particles show the same proclivity of terminase to orient with its central pore roughly perpendicular to the vitreous ice surface. However, the shape of the enzyme is notably different (Figure 5). The 2D classes are more uniform and show a much more compact and well-defined molecule. An overwhelming majority of the particles have four out of five large terminase arms pulled in over the small terminase ring. The fifth TerL subunit appears extended with weak fuzzy density, consistent with considerable freedom of motion for this subunit, which may also be less tightly bound as evidenced in the AUC and CDMS data. Thus, the overall pentameric molecule deviates from strict 5-fold symmetry in a 4 + 1 fashion. We interpret this to indicate that four large terminase subunits tightly interact with each other forming an open TerL ring of a DNA packaging motor. Presumably, this represents the ATP-mediated tight DNA-binding conformation observed biochemically (5,67). The ATPyS-driven interactions between the four TerL subunits most likely maintain the integrity of three inter-subunit ATPase active sites per motor assembly. Such an interpretation is consistent with the structure of the phi29 motor stalled with ATPγS (68) and the kinetic results described below.

### Predicted structure of the terminase protomer

We used AI, machine learning-based structure prediction tools, including different implementations of Alphafold2 and Rosettafold, to predict the quaternary structure of the terminase protomer (60,69,70). The structures predicted by the different programs varied considerably and should of course be interpreted with caution. The most reasonable structures were produced with the full implementation of Alphafold2 installed at the Texas Advanced Computing Center.

Small terminase is predicted as a predominantly α-helical protein consisting of three domains (Figure 7A). The N-terminal domain in the computed structure models (CSMs) is a DNA-binding winged helix-turn-helix (wHTH) domain that is essentially identical to the previously determined NMR structure (Figure 7B) (71). The C-terminal part of the TerS dimer is folded into a four-helix bundle, which interacts with the N-terminal α-helix of TerL, and thus this domain likely plays a key role in protomer assembly (Figure 7C). Such an arrangement agrees with biochemical and genetic data showing that TerS and TerL oligomerize into the protomer via C-terminal and N-terminal structural motifs, respectively (72). The wHTH and four-helix bundle oligomerization domains are integrated into the TerS structure by a pair of α-helixes and two sets of flexible linkers (Figure 7A). Though the confidence of the TerS prediction in the CSMs of the terminase protomer is only moderate, the computed structures agree well with accumulated experimental structural and functional data, both presented here (see below) and previously reported (5,72).

The two domains of TerL, ATPse and nuclease, are predicted with high confidence and there is little variation in these regions in CSMs (Figure 7A). A Dali search performed with individual domains of TerL returned the crystal structure of large terminase of bacteriophage HK97 (6z6d) as the closest homolog (73,74). Residues 29–366 of lambda TerL, comprising the whole ATPase domain, align with residues 6–272 of TerL of HK97 with RMSD of 2.7 Å. The lambda ATPase domain in CSMs is very similar to HK97 with the notable exception of a large, predominantly β-structural, insertion consisting of residues 239–319 (Figure 7D). The lambda nuclease domain diverges more from the HK97 homolog, with an RMSD of 3.1 Å over residues equivalent to 373–623 in the lambda TerL structure (Figure 7E).

### ATPase activity

We performed a kinetic analysis of lambda terminase ATP hydrolysis activity. As previously demonstrated, the terminase protomer is devoid of ATPase activity, while the assembled ring efficiently hydrolyzes ATP (Figure 7, *inset*). We analyzed the kinetic data according to the Michaelis-Menten model, which yielded *K_M_*= 0.495 ± 0.070 μM, *V_M_*= 0.157 ± 0.006 μM/min and *k_cat_*= 15.7 ± 0.6 min^−1^. We note that the *K_M_* obtained here is an order of magnitude lower than that previously reported (61,75). Nucleotide-binding sites typically reside at the subunit interfaces in oligomeric ATPase enzymes and nucleotide binding and complex assembly are thus thermodynamically linked (76). We propose that the high affinity reported here reflects nucleotide binding to the pre-assembled active sites formed between adjacent subunits in the ring.

## Discussion

AUC and SAXS analysis are consistent with a holoenzyme that is a single species in solution with a molecular weight of ∼514 kDa, corresponding to a complex composed of ∼ 4.5 protomers. The SAXS data further indicate that the protomers are compact and mostly well-folded and form a conical assembly. Mass spectrometry shows that the holoenzyme complex is composed of five protomers with the additional nuance that in a minority of particles, one of the TerL subunits appears to have dissociated. Classification of 2D cryoEM images indicates that the particles resemble a starfish, with five flexibly extended TerL subunits radiating from a TerS central disk. There is no density in the center of the disk, and a central channel of ∼29 Å. The radial extensions are related by a ∼72-degree rotation; however, density for one or more of the extended arms is weaker, diffuse and/or substantially differently positioned in most 2D class averages. The most parsimonious explanation consistent with the aggregate data is that the terminase holoenzyme ring is composed of five protomers [(TerL•TerS_2_)_5_], but that one TerL subunit is flexible and arranged differently than the others. Such an arrangement, along with the non-trivial shape of the particle, might explain the non-integer number of subunits observed in AUC and SAXS experiments.

Based on our data and published structures of TerS ring complexes from other phage systems, we propose that the overall conical structure of holoenzyme is organized by ten TerS subunits, donated by five protomers [(TerL•TerS_2_)_5_], arranged as a ring at the narrow end of the cone. Of note, the central diameter of the ring (∼29 Å) can accommodate duplex DNA. We further propose that the five TerL subunits are arranged around the wide end of the cone to form the observed starfish structure in an asymmetric 4 + 1 fashion with four of the subunits being mostly radially extended and one retracted (Figure 5). CryoEM class averages show that ATPγS binding induces a more compact conformation, wherein four of the TerL subunits form an open ring while the fifth one is unengaged and thus flexible. Hence, the nucleotide-bound complex retains the overall 4 + 1 arrangement of the TerL subunits. Presumably, ATP-binding causes the open TerL ring to ‘clamp down’ on the DNA duplex. This is consistent with the observation that ATP increases the affinity of lambda terminase for *cos* DNA (30) and with the ‘open’ and ‘closed’ conformations observed in negative stained micrographs previously reported from our lab (25,27).

The data reported here are commensurate with the known structures of packaging motors assembled from isolated TerL subunits (5,36,77) and suggest that λ terminase holoenzyme similarly assembles a pentameric motor arranged in a parallel orientation, poised for unidirectional translocation of DNA. We propose that the proximate TerL ring binds to the portal and to DNA to physically translocate the duplex into the capsid, while the downstream TerS ring is positioned to act as a ‘sliding clamp’, analogous to those involved in DNA replication (78), to ensure a highly processive machine. In this fashion, DNA passes through a decameric TerS ring, a pentameric TerL ring (the actual motor), and finally a dodecameric portal ring *en route* to the capsid interior.

While the data presented herein are consistent with that expected of a *bone fide* motor complex, they differ significantly from that proposed for the maturation complex (*vide supra*). This raises the question as to how a necessary head-to-head dimer of dimers in a maturation complex might transition to a parallel, pentameric packaging motor complex. We previously proposed a ‘symmetry resolution’ model to address this conundrum (4,5) and the data presented herein provides additional insight and suggest an alternative model for the nuclease complex. In this model, *five* terminase protomers assemble at the *cosB* subsite resulting in a decameric TerS ring that encircles the duplex. Terminase assembly on *cos* DNA is strongly stimulated by ATP (30) and ATP binding drives four of the TerL subunits into second ring (Figure 5) that similarly encircles the duplex and positions one of the TerL nuclease domains at the downstream nicking site (Figure 8). While there are five TerL subunits in the complex, they are not arranged as a ring with strict C5 symmetry; one subunit is bound less tightly, thus conferring significant positional flexibility and resulting in an incomplete ring. Further, both secondary structure prediction and the predicted structure of the lambda protomer indicate that the linker between the N- and C-terminal domains of TerL confers additional flexibility of the C-terminal nuclease domain, allowing it to rotate ∼180° and bind to the symmetric upstream nicking site. This effectively forms a head-to-head nuclease dimer capable of nicking the palindromic duplex. Presumably, subsequent strand separation activity of terminase ejects the upstream DNA fragment (*cos*-cleavage reaction) (28,31), resulting in a stable post-cleavage complex ready to bind to the dodecameric portal vertex.

It has been shown that prior to binding a procapsid, the post-cleavage complex has down-regulated ATPase activity and is extremely stable (*T*_1/2_∼ 8 h) (79,80). A lingering puzzling question is what prevents this complex from prematurely hydrolyzing ATP and translocating DNA before the complex binds to the procapsid. We propose that the stability of the complex is derived from TerS binding to *cosB* and from TerL binding to the newly formed genome end, which locks the motor subunits into a catalytically inactive configuration. The complex is stabilized by both (i) the decameric TerS ring structure circumscribing DNA, an arrangement that is consistent with the published structures of TerS assemblies (*vide supra*); and by (ii) the open tetrameric TerL ring bound to, and protecting, the genome end. We further propose that the lack of ATPase activity is further inhibited by the deviation from 5-fold symmetry in the post-cleavage complex. This 4 + 1 geometry observed in the substrate-bound state effectively excludes one of the TerL ATPase subunits from the ring (Figures 6 and 8), thus poisoning cooperative ATP hydrolysis required for motor function. Such an interpretation is consistent with our biochemical data demonstrating that (i) the ATPase activity of the enzyme is downregulated in this complex (80) and that (ii) a single defective ATPase subunit in the motor complex abrogates DNA packaging activity (38). Upon binding to the portal vertex of an empty procapsid, TerL subunits to adopt true 5-fold symmetry, activating all five ATPase catalytic sites for cooperative ATP hydrolysis, triggering ‘*cos*-clearance’ and initiating DNA packaging (Figure 8). The proposed model integrates direct visualization of λ terminase holoenzyme in native conditions with decades of genetic, biochemical, biophysical, and structural characterization of the terminase maturation and motor complexes, and illuminates the transition between maturation and translocation. Further, the model is harmonious with both well characterized head-to-head nuclease complexes and the well characterized pentameric symmetry of phage motor complexes. Importantly, in our model, a single pentameric complex catalyzes the two disparate packaging functions with minimal conformational reorganization. This streamlines the transitions between maturation and translocation to allow efficient processive genome packaging from the concatemeric DNA substrate.

## Data Availability

The data underlying this article are available in the article and in its online supplementary material. The original scan of the gels will be shared on reasonable request to the corresponding author. Kinetic analysis of the terminase protomer and the assembled holoenzyme. *Inset:* Time course for ADP formation. Dashed line represents protomer data while solid lines represent data for the pentamer ring. Only the linear portions of the data were used to calculate the observed rates. Main figure shows a Michaelis–Menten plot; solid line represents the best fit of the data. Modeling the terminase protomer. Panel **A**. Four best Alphafold 2 computed structure models (CSMs) of the terminase protomer superimposed and colored according to the confidence of prediction (pLDDT score). Panel **B**. Comparison of the best model of the DNA-binding domain with the experimentally determined NMR structure (PDB accession number 1j9i). Numbering of the α-helixes is shown starting from the N-terminus of TerS. The CSM is colored according to the pLDDT score and the NMR structure is shown in grey. Panel **C**. The oligomerization domain of small terminase anchors the N-terminal α-helix of large teminase. Numbering of the α-helixes and the range of displayed residues are shown. Panel **D**. Superposion of the TerL ATPse domain of lambda and the ATPase of the HK97 terminase, the closest Dali hit (PDB accession number 6z6d). Panel **E**. Superposion of the TerL nuclease domains of lambda and HK97. The lambda CSMs on panels D and E are colored according to pLDDT score and the HK97 structure is shown in grey. Model for Symmetry Resolution. Non-engaged nuclease subunits are omitted for clarity in panels 1, 2 and 3. Details provided in the text.

## References

[1] Knipe D.M. , Howley P.M. Fields Virology. 2007; 5th edn NY Lippincott-Williams, and Wilkins.

[2] Roizman B. , Knipe D.M., Whitley R.J. Knipe D.M. , Howley P.M. Fields Virology. 2007; 5th edn. NY Lippincott, Williams, and Wilkins 2501–2602.

[3] Calendar R. , Abedon S.T. The Bacteriophages. 2006; NY Oxford University Press.

[4] Catalano C.E. Bamford D.H. , Zuckerman M. Encyclopedia of Virology (Fourth Edition). 2021; Oxford Academic Press 124–135.

[5] Catalano C.E. , Morais M.C. Viral Genome Packaging Machines: Structure and Enzymology. 2021; Cambridge, MA Academic Press. 10.1016/bs.enz.2021.09.006 34861943

[6] Casjens S.R. The DNA-packaging nanomotor of tailed bacteriophages. Nat. Rev. Micro. 2011; 9:647–657. 10.1038/nrmicro2632 21836625

[7] Rao V.B. , Feiss M. Mechanisms of DNA packaging by large double-stranded DNA viruses. Annu. Rev. Virol. 2015; 2:351–378. 26958920 10.1146/annurev-virology-100114-055212 PMC4785836

[8] Catalano C.E. Catalano C.E. Viral Genome Packaging Machines: Genetics, Structure, and Mechanism. 2005; NY Springer 1–4.

[9] Al-Zahrani A.S. , Kondabagil K., Gao S., Kelly N., Ghosh-Kumar M., Rao V.B. The small terminase, gp16, of bacteriophage T4 is a regulator of the DNA packaging motor. J. Biol. Chem. 2009; 284:24490–24500. 19561086 10.1074/jbc.M109.025007 PMC2782041

[10] Feiss M. , Rao B.N. Rossmann M.G. , Rao B.N. Viral Molecular Machines. 2012; Springer US 498–509.

[11] Pajak J. , Dill E., Reyes-Aldrete E., White M.A., Kelch B.A., Jardine P.J., Arya G., Morais M.C. Atomistic basis of force generation, translocation, and coordination in a viral genome packaging motor. Nucleic Acids Res. 2021; 49:6474–6488. 34050764 10.1093/nar/gkab372 PMC8216284

[12] Reyes-Aldrete E. , Dill E.A., Bussetta C., Szymanski M.R., Diemer G., Maindola P., White M.A., Bujalowski W.M., Choi K.H., Morais M.C. Biochemical and biophysical characterization of the dsDNA packaging motor from the *Lactococcus lactis* bacteriophage Asccphi28. Viruses. 2020; 13:15. 33374840 10.3390/v13010015 PMC7823558

[13] Hayes J.A. , Kelch B.A. Bamford D.H. , Zuckerman M. Encyclopedia of Virology (Fourth Edition). 2021; Oxford Academic Press 148–159.

[14] Yang Q. , Berton N., Manning M.C., Catalano C.E. Domain structure of gpNu1, a phage lambda DNA packaging protein. Biochemistry. 1999; 38:14238–14247. 10571997 10.1021/bi991408f

[15] Kondabagil K.R. , Rao V.B. A critical coiled coil motif in the small terminase, gp16, from bacteriophage T4: insights into DNA packaging initiation and assembly of packaging motor. J. Mol. Biol. 2006; 358:67–82. 16513134 10.1016/j.jmb.2006.01.078

[16] Lin H. , Simon M.N., Black L.W. Purification and characterization of the small subunit of phage T4 terminase, gp16, required for DNA packaging. J. Biol. Chem. 1997; 272:3495–3501. 9013596 10.1074/jbc.272.6.3495

[17] Zhao H. , Finch C.J., Sequeira R.D., Johnson B.A., Johnson J.E., Casjens S.R., Tang L. Crystal structure of the DNA-recognition component of the bacterial virus Sf6 genome-packaging machine. Proc. Natl. Acad. Sci. 2010; 107:1971–1976. 20133842 10.1073/pnas.0908569107 PMC2836615

[18] Büttner C.R. , Chechik M., Ortiz-Lombardía M., Smits C., Ebong I.-O., Chechik V., Jeschke G., Dykeman E., Benini S., Robinson C.V. et al. Structural basis for DNA recognition and loading into a viral packaging motor. Proc. Natl. Acad. Sci. U.S.A. 2012; 109:811–816. 22207627 10.1073/pnas.1110270109 PMC3271932

[19] Sun S. , Gao S., Kondabagil K., Xiang Y., Rossmann M.G., Rao V.B. Structure and function of the small terminase component of the DNA packaging machine in T4-like bacteriophages. Proc. Natl. Acad. Sci. U.S.A. 2012; 109:817–822. 22207623 10.1073/pnas.1110224109 PMC3271864

[20] Roy A. , Bhardwaj A., Datta P., Lander G.C., Cingolani G. Small terminase couples viral DNA binding to genome-packaging ATPase activity. Structure. 2012; 20:1403–1413. 22771211 10.1016/j.str.2012.05.014 PMC3563279

[21] Zhao H. , Kamau Y.N., Christensen T.E., Tang L. Structural and functional studies of the Phage Sf6 terminase small subunit reveal a DNA-spooling device facilitated by structural plasticity. J. Mol. Biol. 2012; 423:413–426. 22858866 10.1016/j.jmb.2012.07.016 PMC3572731

[22] McNulty R. , Lokareddy R.K., Roy A., Yang Y., Lander G.C., Heck A.J.R., Johnson J.E., Cingolani G. Architecture of the complex formed by large and small terminase subunits from bacteriophage P22. J. Mol. Biol. 2015; 427:3285–3299. 26301600 10.1016/j.jmb.2015.08.013 PMC4587339

[23] Roy A. , Bhardwaj A., Datta P., Lander G.C., Cingolani G. Small terminase couples viral DNA binding to genome-packaging ATPase activity. Structure. 2012; 20:1403–1413. 22771211 10.1016/j.str.2012.05.014 PMC3563279

[24] Tomka M.A. , Catalano C.E. Physical and kinetic characterization of the DNA packaging enzyme from bacteriophage lambda. J. Biol. Chem. 1993; 268:3056–3065. 8428984

[25] Maluf N.K. , Yang Q., Catalano C.E. Self-association properties of the bacteriophage lambda terminase holoenzyme: implications for the DNA packaging motor. J. Mol. Biol. 2005; 347:523–542. 15755448 10.1016/j.jmb.2005.01.016

[26] Maluf N.K. , Gaussier H., Bogner E., Feiss M., Catalano C.E. Assembly of bacteriophage lambda terminase into a viral DNA maturation and packaging machine. Biochemistry. 2006; 45:15259–15268. 17176048 10.1021/bi0615036

[27] Yang T.-C. , Ortiz D., Nosaka L.A., Lander G.C., Catalano C.E. Thermodynamic interrogation of the assembly of a viral genome packaging motor complex. Biophys. J. 2015; 109:1663–1675. 26488657 10.1016/j.bpj.2015.08.037 PMC4624345

[28] Yang Q. , Catalano C.E. Kinetic characterization of the strand separation (“helicase”) activity of the DNA packaging enzyme from bacteriophage lambda. Biochemistry. 1997; 36:10638–10645. 9271494 10.1021/bi970689t

[29] Yang Q. , Catalano C.E. ATP serves as a nucleotide switch coupling the genome maturation and packaging motor complexes of a virus assembly machine. Nucleic Acids Res. 2020; 48:5006–5015. 32255177 10.1093/nar/gkaa205 PMC7229814

[30] Yang Q. , Catalano C.E. A minimal kinetic model for a viral DNA packaging machine. Biochemistry. 2004; 43:289–299. 14717582 10.1021/bi035532h

[31] Chang J.R. , Andrews B.T., Catalano C.E. Energy independent helicase activity of a viral genome packaging. Biochemistry. 2012; 51:391–400. 22191393 10.1021/bi201604b PMC3266165

[32] Krüger D.H. , Barcak G.J., Reuter M., Smith H.O. EcoRII can be activated to cleave refractory DNA recognition sites. Nucleic Acids Res. 1988; 16:3997–4008. 2836807 10.1093/nar/16.9.3997 PMC336570

[33] Pingoud A. , Fuxreiter M., Pingoud V., Wende W. Type II restriction endonucleases: structure and mechanism. Cell. Mol. Life Sci. 2005; 62:685–707. 15770420 10.1007/s00018-004-4513-1 PMC11924531

[34] Chan S.-H. , Stoddard B.L., Xu S.-Y. Natural and engineered nicking endonucleases— from cleavage mechanism to engineering of strand-specificity. Nucleic Acids Res. 2011; 39:1–18. 20805246 10.1093/nar/gkq742 PMC3017599

[35] Mao H. , Saha M., Reyes-Aldrete E., Sherman M.B., Woodson M., Atz R., Grimes S., Jardine P.J., Morais M.C Structural and molecular basis for coordination in a viral DNA packaging motor. Cell Rep. 2016; 14:2017–2029. 26904950 10.1016/j.celrep.2016.01.058 PMC4824181

[36] Woodson M. , Pajak J., Mahler B.P., Zhao W., Zhang W., Arya G., White M.A., Jardine P.J., Morais M.C. A viral genome packaging motor transitions between cyclic and helical symmetry to translocate dsDNA. Sci. Adv. 2021; 7:eabc1955. 33962953 10.1126/sciadv.abc1955 PMC8104870

[37] Mahler B.P. , Bujalowski P.J., Mao H., Dill E.A., Jardine P.J., Choi K.H., Morais M.C. NMR structure of a vestigial nuclease provides insight into the evolution of functional transitions in viral dsDNA packaging motors. Nucleic Acids Res. 2020; 48:11737–11749. 33089330 10.1093/nar/gkaa874 PMC7672431

[38] Andrews B.T. , Catalano C.E. Strong subunit coordination drives a powerful viral DNA packaging motor. Proc. Natl. Acad. Sci. U.S.A. 2013; 110:5909–5914. 23530228 10.1073/pnas.1222820110 PMC3625343

[39] Yang T.-C. , Ortiz D., Yang Q., Angelis R.D., Sanyal S.J., Catalano C.E. Physical and functional characterization of nucleoprotein complexes along a viral assembly pathway. Biophys. J. 2017; 112:1551–1560. 28445747 10.1016/j.bpj.2017.02.041 PMC5406279

[40] Yang Q. , Catalano C.E. ATP serves as a nucleotide switch coupling the genome maturation and packaging motor complexes of a virus assembly machine. Nucleic Acids Res. 2020; 48:5006–5015. 32255177 10.1093/nar/gkaa205 PMC7229814

[41] Hura G.L. , Menon A.L., Hammel M., Rambo R.P., Poole Ii F.L., Tsutakawa S.E., Jenney F.E. Jr, Classen S., Frankel K.A., Hopkins R.C. et al. Robust, high-throughput solution structural analyses by small angle X-ray scattering (SAXS). Nat. Methods. 2009; 6:606–612. 19620974 10.1038/nmeth.1353 PMC3094553

[42] Konarev P.V. , Volkov V.V., Petoukhov M.V., Svergun D.I. ATSAS 2.1, a program package for small-angle scattering data analysis. J. Appl. Crystallogr. 2006; 39:277–286. 10.1107/S0021889812007662 PMC4233345 25484842

[43] Konarev P.V. , Volkov V.V., Sokolova A.V., Koch M.H.J., Svergun D.I. PRIMUS: a Windows PC-based system for small-angle scattering data analysis. J. Appl. Crystallogr. 2003; 36:1277–1282.

[44] Svergun D. Determination of the regularization parameter in indirect-transform methods using perceptual criteria. J. Appl. Crystallogr. 1992; 25:495–503.

[45] Mertens H.D.T. , Svergun D.I. Structural characterization of proteins and complexes using small-angle X-ray solution scattering. J. Struct. Biol. 2010; 172:128–141. 20558299 10.1016/j.jsb.2010.06.012

[46] Porod G. Glatter O. Small Angle X-ray Scattering. 1982; London, UK Academic Press.

[47] Svergun D.I. , Petoukhov M.V., Koch M.H.J. Determination of domain structure of proteins from X-ray solution scattering. Biophys. J. 2001; 80:2946–2953. 11371467 10.1016/S0006-3495(01)76260-1 PMC1301478

[48] Schuck P. On the analysis of protein self-association by sedimentation velocity analytical ultracentrifugation. Anal. Biochem. 2003; 320:104–124. 12895474 10.1016/s0003-2697(03)00289-6

[49] Stafford W.F. , Sherwood P.J. Uchiyama S. , Arisaka F., Stafford W.F., Laue T. Analytical Ultracentrifugation: Instrumentation, Software, and Applications. 2016; Tokyo Springer Japan 103–117.

[50] Stafford W.F. , Sherwood P.J. Analysis of heterologous interacting systems by sedimentation velocity: curve fitting algorithms for estimation of sedimentation coefficients, equilibrium and kinetic constants. Biophys. Chem. 2004; 108:231–243. 15043932 10.1016/j.bpc.2003.10.028

[51] Contino N.C. , Jarrold M.F. Charge detection mass spectrometry for single ions with a limit of detection of 30 charges. Int. J. Mass spectrom. 2013; 345-347:153–159.

[52] Keifer D.Z. , Shinholt D.L., Jarrold M.F. Charge detection mass spectrometry with almost perfect charge accuracy. Anal. Chem. 2015; 87:10330–10337. 26418830 10.1021/acs.analchem.5b02324

[53] Hogan J.A. , Jarrold M.F. Optimized electrostatic linear ion trap for charge detection mass spectrometry. J. Am. Soc. Mass. Spectrom. 2018; 29:2086–2095. 29987663 10.1007/s13361-018-2007-x

[54] Draper B.E. , Anthony S.N., Jarrold M.F. The FUNPET—a new hybrid ion funnel-ion carpet atmospheric pressure interface for the simultaneous transmission of a broad mass range. J. Am. Soc. Mass. Spectrom. 2018; 29:2160–2172. 30112619 10.1007/s13361-018-2038-3

[55] Draper B.E. , Jarrold M.F. Real-time analysis and signal optimization for charge detection mass spectrometry. J. Am. Soc. Mass. Spectrom. 2019; 30:898–904. 30993638 10.1007/s13361-019-02172-z

[56] Todd A.R. , Alexander A.W., Jarrold M.F. Implementation of a charge-sensitive amplifier without a feedback resistor for charge detection mass spectrometry reduces noise and enables detection of individual ions carrying a single charge. J. Am. Soc. Mass. Spectrom. 2020; 31:146–154. 32881508 10.1021/jasms.9b00010

[57] Todd A.R. , Jarrold M.F. Dynamic calibration enables high-accuracy charge measurements on individual ions for charge detection mass spectrometry. J. Am. Soc. Mass. Spectrom. 2020; 31:1241–1248. 32353231 10.1021/jasms.0c00081

[58] Todd A.R. , Barnes L.F., Young K., Zlotnick A., Jarrold M.F. Higher resolution charge detection mass spectrometry. Anal. Chem. 2020; 92:11357–11364. 32806905 10.1021/acs.analchem.0c02133 PMC8587657

[59] Punjani A. , Rubinstein J.L., Fleet D.J., Brubaker M.A. cryoSPARC: algorithms for rapid unsupervised cryo-EM structure determination. Nat. Methods. 2017; 14:290–296. 28165473 10.1038/nmeth.4169

[60] Jumper J. , Evans R., Pritzel A., Green T., Figurnov M., Ronneberger O., Tunyasuvunakool K., Bates R., Zidek A., Potapenko A. et al. Highly accurate protein structure prediction with AlphaFold. Nature. 2021; 596:583–589. 34265844 10.1038/s41586-021-03819-2 PMC8371605

[61] Tomka M.A. , Catalano C.E. Kinetic characterization of the ATPase activity of the DNA packaging enzyme from bacteriophage lambda. Biochemistry. 1993; 32:11992–11997. 8218275 10.1021/bi00096a008

[62] Woods L. , Catalano C.E. Kinetic characterization of the GTPase activity of phage lambda terminase: evidence for communication between the two “NTPase” catalytic sites of the enzyme. Biochemistry. 1999; 38:14624–14630. 10545186 10.1021/bi990866l

[63] Volkov V.V. , Svergun D.I. Uniqueness of ab initio shape determination in small-angle scattering. J. Appl. Crystallogr. 2003; 36:860–864. 10.1107/S0021889809000338 PMC5023043 27630371

[64] Putnam C.D. , Hammel M., Hura G.L., Tainer J.A. X-ray solution scattering (SAXS) combined with crystallography and computation: defining accurate macromolecular structures, conformations and assemblies in solution. Q. Rev. Biophys. 2007; 40:191–285. 18078545 10.1017/S0033583507004635

[65] Ortega A. , Amorós D., de la Torre J.G. Prediction of hydrodynamic and other solution properties of rigid proteins from atomic- and residue-level models. Biophys. J. 2011; 101:892–898. 21843480 10.1016/j.bpj.2011.06.046 PMC3175065

[66] Morais M.C. The dsDNA packaging motor in bacteriophage ø29. Adv. Exp. Med. Biol. 2012; 726:511–547. 22297529 10.1007/978-1-4614-0980-9_23

[67] delToro D. , Ortiz D., Ordyan M., Pajak J., Sippy J., Catala A., Oh C.S., Vu A., Arya G., Smith D.E. et al. Functional dissection of a viral DNA packaging machine's walker B motif. J. Mol. Biol. 2019; 431:4455–4474. 31473160 10.1016/j.jmb.2019.08.012 PMC7416571

[68] Woodson M. , Pajak J., Mahler B.P., Zhao W., Zhang W., Arya G., White M.A., Jardine P.J., Morais M.C. A viral genome packaging motor transitions between cyclic and helical symmetry to translocate dsDNA. Sci. Adv. 2021; 7:eabc1955. 33962953 10.1126/sciadv.abc1955 PMC8104870

[69] Baek M. , DiMaio F., Anishchenko I., Dauparas J., Ovchinnikov S., Lee G.R., Wang J., Cong Q., Kinch L.N., Schaeffer R.D. et al. Accurate prediction of protein structures and interactions using a three-track neural network. Science. 2021; 373:871–876. 34282049 10.1126/science.abj8754 PMC7612213

[70] Mirdita M. , Schutze K., Moriwaki Y., Heo L., Ovchinnikov S., Steinegger M. ColabFold: making protein folding accessible to all. Nat. Methods. 2022; 19:679–682. 35637307 10.1038/s41592-022-01488-1 PMC9184281

[71] de Beer T. , Fang J., Ortega M., Yang Q., Maes L., Duffy C., Berton N., Sippy J., Overduin M., Feiss M. et al. Insights into specific DNA recognition during the assembly of a viral genome packaging machine. Mol. Cell. 2002; 9:981–991. 12049735 10.1016/s1097-2765(02)00537-3

[72] Catalano C.E. The terminase enzyme from bacteriophage lambda: a DNA-packaging machine. Cell. Mol. Life Sci. 2000; 57:128–148. 10949585 10.1007/s000180050503 PMC11147104

[73] Fung H.K.H. , Grimes S., Huet A., Duda R.L., Chechik M., Gault J., Robinson C.V., Hendrix R.W., Jardine P.J., Conway J.F. et al. Structural basis of DNA packaging by a ring-type ATPase from an archetypal viral system. Nucleic Acids Res. 2022; 50:8719–8732. 35947691 10.1093/nar/gkac647 PMC9410871

[74] Holm L. , Rosenstrom P. Dali server: conservation mapping in 3D. Nucleic Acids Res. 2010; 38:W545–W549. 20457744 10.1093/nar/gkq366 PMC2896194

[75] Hwang Y. , Catalano C.E., Feiss M. Kinetic and mutational dissection of the two ATPase activities of terminase, the DNA packaging enzyme of bacteriophage Chi. Biochemistry. 1996; 35:2796–2803. 8611586 10.1021/bi952322z

[76] White S.R. , Lauring B. AAA+ ATPases: achieving diversity of function with conserved machinery. Traffic. 2007; 8:1657–1667. 17897320 10.1111/j.1600-0854.2007.00642.x

[77] Pajak J. , Atz R., Hilbert B.J., Morais M.C., Kelch B.A., Jardine P.J., Arya G. Viral packaging ATPases utilize a glutamate switch to couple ATPase activity and DNA translocation. Proc. Natl. Acad. Sci. U.S.A. 2021; 118:e2024928118. 33888587 10.1073/pnas.2024928118 PMC8092589

[78] Moldovan G.L. , Pfander B., Jentsch S. PCNA, the maestro of the replication fork. Cell. 2007; 129:665–679. 17512402 10.1016/j.cell.2007.05.003

[79] Yang Q. , Hanagan A., Catalano C.E. Assembly of a nucleoprotein complex required for DNA packaging by bacteriophage lambda. Biochemistry. 1997; 36:2744–2752. 9062101 10.1021/bi9622682

[80] Chang J.R. , Andrews B.T., Catalano C.E. Energy-independent helicase activity of a viral genome packaging motor. Biochemistry. 2012; 51:391–400. 22191393 10.1021/bi201604b PMC3266165

